# Clinical characteristics, etiology, and outcomes of acute pancreatitis at a tertiary referral center in Latvia: a retrospective cohort study

**DOI:** 10.1186/s12876-026-05064-1

**Published:** 2026-06-27

**Authors:** Daniil Varlamov, Abdulrahman Al-Dawoudi, Mujahed Dalain, Nazar Kopytko, Aiga Staka, Davis Freimanis

**Affiliations:** 1 Faculty of Medicine and Life Sciences, University of Latvia, Riga, Latvia; 2 Medical Faculty No. 1, Poltava State Medical University, Poltava, Ukraine; 3 Department of Gastroenterology, Pauls Stradins Clinical University Hospital, Riga, Latvia

**Keywords:** Acute pancreatitis, Alcohol-related pancreatitis, Gallstone pancreatitis, Pancreatitis complications, Epidemiology, Retrospective cohort, Baltic region

## Abstract

**Background:**

Acute pancreatitis is a common cause of gastrointestinal hospitalization, but detailed clinical cohort data from the Baltic region are limited. We aimed to characterize the clinical features, etiologies, comorbidities, and in-hospital outcomes of acute pancreatitis at a tertiary-center in Latvia.

**Methods:**

We conducted a retrospective single-center cohort study of adults hospitalized with acute pancreatitis between 2020 and 2024, including only the index admission for each patient. Cases were identified using ICD-10 codes and confirmed according to the Revised Atlanta Classification. Demographic characteristics, etiologic factors, comorbidities, and clinical outcomes were analyzed. Multivariable logistic regression was used to identify factors independently associated with in-hospital complications.

**Results:**

A total of 1,047 patients were included; the median age was 50 years (interquartile range, IQR 39–64.5), and 62.0% were male. Alcohol-related pancreatitis was the most common etiology (37.0%), followed by gallstone pancreatitis (24.7%). Overall, 215 patients (20.5%) developed at least one in-hospital complication. In multivariable analysis, alcohol-related pancreatitis (Odds ratio, OR 2.63, 95% Confidence interval, CI 1.59–4.36) and other etiologies (OR 2.89, 95% CI 1.79–4.65) were associated with higher odds of complications than gallstone pancreatitis. Diabetes mellitus (OR 1.69, 95% CI 1.10–2.56) and heart failure (OR 1.70, 95% CI 1.01–2.86) were also associated with increased risk. Median length of hospital stay was 6 days (IQR 4–9), intensive care unit admission occurred in 1.4% of patients, and in-hospital mortality was 5.3%.

**Conclusions:**

In this tertiary-center cohort, alcohol-related pancreatitis predominated and was associated with higher odds of the composite in-hospital complication endpoint than gallstone pancreatitis. These findings suggest a region-specific etiologic pattern and indicate that alcohol-related disease may contribute substantially to the acute pancreatitis case burden and adverse in-hospital outcomes in this setting.

**Supplementary Information:**

The online version contains supplementary material available at https://doi.org/10.1186/s12876-026-05064-1.

## Background

Acute pancreatitis (AP) is one of the most common gastrointestinal diseases requiring hospitalization worldwide [1]. The estimated annual incidence ranges from approximately 13 to 45 cases per 100,000 people [2] and has increased over recent decades in many regions, including Europe [3, 4]. This condition is associated with substantial morbidity and inpatient healthcare resource utilization [5, 6].

In European populations, gallstone disease and alcohol use account for most cases, although their relative contributions vary across countries [4, 7, 8]. The underlying cause is clinically important because it influences patient demographics, recurrence risk, and patterns of disease presentation observed in hospitalized cohorts [4, 8]. Etiological differences are also associated with variations in complication profiles and short-term outcomes, highlighting the importance of cause-specific characterization in clinical studies of pancreatitis [6, 9]. Among the most severe forms of the disease, persistent organ failure and local complications such as pancreatic necrosis are associated with adverse outcomes [10].

Despite standardized diagnostic criteria established by the Revised Atlanta Classification [10], substantial heterogeneity persists in the clinical description and reporting of acute pancreatitis across healthcare systems [4]. While large epidemiological studies have focused on incidence and mortality, detailed analyses of clinical presentation, comorbidity burden, and in-hospital outcomes are primarily derived from institutional cohorts [3, 4, 6]. Several such cohorts describing clinical characteristics and outcomes of the disease have been reported from different European regions [6, 11]. However, detailed institutional data describing the clinical characteristics and outcomes of acute pancreatitis remain scarce in the Baltic region, including Latvia, where evidence is largely limited to population-level analyses [4]. This gap is notable given evidence that Eastern Europe has one of the highest burdens of alcohol-related pancreatitis globally [12, 13].

The aim of this study was to describe the clinical characteristics, etiological distribution, comorbidity burden, and in-hospital outcomes of adults hospitalized with acute pancreatitis at a tertiary referral center in Latvia over a five-year period.

## Methods

### Study design and setting

This was a retrospective single-center cohort study conducted at Pauls Stradins Clinical University Hospital, a tertiary referral center in Riga, Latvia. The study included consecutive adult patients hospitalized with acute pancreatitis between 2020 and 2024. Reporting followed the Strengthening the Reporting of Observational Studies in Epidemiology (STROBE) guidelines for cohort studies, and a completed STROBE checklist is provided as Supporting Information File S1.

### Study population and eligibility criteria

Hospital records were screened to identify all adult hospitalizations (≥ 18 years) between 2020 and 2024 with a discharge diagnosis of acute pancreatitis (International Classification of Diseases, 10th Revision (ICD-10) code K85). All identified hospitalizations were individually reviewed to confirm fulfillment of diagnostic criteria according to the Revised Atlanta Classification, requiring at least two of the following three criteria: acute abdominal pain consistent with pancreatitis; serum amylase and/or lipase ≥ 3 times the upper limit of normal; or characteristic imaging findings [10].

To avoid overrepresentation, only the first hospitalization (index admission) was included, and subsequent admissions were excluded. Additional exclusion criteria were chronic pancreatitis without an acute episode and insufficient documentation for etiologic classification or in-hospital outcomes.

### Data collection, definitions and variables

Clinical data were collected retrospectively from electronic medical records. The extracted variables included demographic characteristics (age at admission and sex), etiological classification of acute pancreatitis, clinical course variables (intensive care unit (ICU) admissions, length of hospital stay in days, and discharge status), and in-hospital outcomes including local and systemic complications.

Comorbidities were recorded as binary variables and included diabetes mellitus, hypertension, chronic liver disease, chronic kidney disease, and heart failure. In-hospital mortality was derived from the discharge records.

Chronic liver disease was identified from routinely recorded ICD-10 diagnoses and available clinical documentation, including documented cirrhosis, chronic hepatitis, and imaging-based evidence of hepatic steatosis. This variable was therefore treated as a broad comorbidity category rather than as a marker of advanced liver disease alone.

In routine institutional practice, transabdominal ultrasound is used as first-line imaging in patients admitted with acute pancreatitis to assess for gallstones, biliary sludge, cholecystitis, common bile duct dilatation, and other evidence of biliary obstruction. When ultrasound is unavailable, technically limited, or insufficient for diagnostic assessment, computed tomography (CT) is used. In patients with a cholestatic clinical or biochemical pattern, suspected choledocholithiasis, biliary duct dilatation, or visible stones on first-line imaging, additional biliary imaging with magnetic resonance imaging/magnetic resonance cholangiopancreatography (MRI/MRCP) is performed, and ERCP is used when therapeutic duct clearance is indicated. This approach is consistent with guideline recommendations for the evaluation of biliary etiology in acute pancreatitis [8, 14].

Patients were categorized into four etiological groups: alcohol-related, biliary (gallstone), idiopathic, and “other” pancreatitis. The “other” category included less common etiologies such as post-endoscopic retrograde cholangiopancreatography (post-ERCP), drug-induced, infectious, etc.

Etiological classification was based on documented clinical history, alcohol exposure, laboratory findings, imaging results, procedure history, and final discharge diagnosis recorded by the treating team. Idiopathic pancreatitis was assigned when routine inpatient clinical, biochemical, and imaging evaluation did not identify alcohol-related, biliary, metabolic, post-procedural, drug-related, infectious, or other specific causes. Because final discharge coding is performed in routine clinical practice and does not require granular etiological subclassification for all cases in local settings, some less common etiologies were coded under broader acute pancreatitis categories. Therefore, the “other” category was treated as a heterogeneous group.

Local pancreatic complications were defined using Revised Atlanta Classification and included necrotizing pancreatitis, infected necrosis, pancreatic pseudocyst, and walled-off necrosis [10]. Systemic complications were defined as multiple organ dysfunction syndrome (MODS), involving ≥ 2 organ systems and assessed using the Modified Marshall scoring system [15].

### Statistical analysis

#### Descriptive and univariable analyses

Statistical analyses were performed using IBM SPSS Statistics, version 29.0 (IBM Corp., Armonk, NY, USA). Categorical variables are presented as counts and percentages, and continuous variables as mean ± standard deviation (SD) or median (interquartile range, IQR) when non-normally distributed. Normality was assessed using the Shapiro–Wilk test.

Continuous variables were compared using the Mann–Whitney U test (two groups) or Kruskal–Wallis test (> 2 groups). Categorical variables were compared using the chi-square test or Fisher’s exact test when the expected cell count was < 5.

#### Multivariable and sensitivity analyses

Multivariable logistic regression was performed to identify factors associated with in-hospital complications. The primary outcome was a composite of ≥ 1 local complication (necrotizing pancreatitis, infected necrotizing pancreatitis, pancreatic pseudocyst, or walled-off necrosis) or systemic complication (MODS). ICU admission was analyzed separately in a predefined sensitivity analysis.

Variables were selected a priori based on clinical relevance and prior literature [16]. A composite endpoint was used to capture clinically relevant adverse in-hospital events, given the low frequency of individual severe outcomes.

Predictors included age, sex, comorbidities (diabetes mellitus, hypertension, chronic liver disease, chronic kidney disease, and heart failure), and etiology. Age was modeled as a continuous variable and categorical predictors were entered as binary indicators. Gallstone pancreatitis was used as the reference category.

To assess potential effects of the coronavirus disease 2019 (COVID-19) pandemic, admissions during 2020–2021 were compared with those in 2022–2024 using univariable and multivariable analyses.

Sensitivity analyses were conducted to evaluate the robustness of the primary model to variations in cohort definition, outcome specification, and etiologic classification. These included: (1) exclusion of pandemic-period admissions; (2) a stricter outcome definition (necrotizing pancreatitis, infected necrosis, MODS, or ICU admission); and (3) combining idiopathic and “other” etiologies.

No correction for multiple comparisons was applied to the univariable comparisons. These analyses were considered descriptive and exploratory, whereas the primary inferential analysis was the predefined multivariable logistic regression model.

The results are reported as odds ratios (ORs) with 95% confidence intervals (CIs). Records with missing key variables were excluded during cohort selection, therefore, no missing data were present in the final dataset. All tests were two-tailed, and *p* < 0.05 considered statistically significant.

## Results

**Fig. 1:**
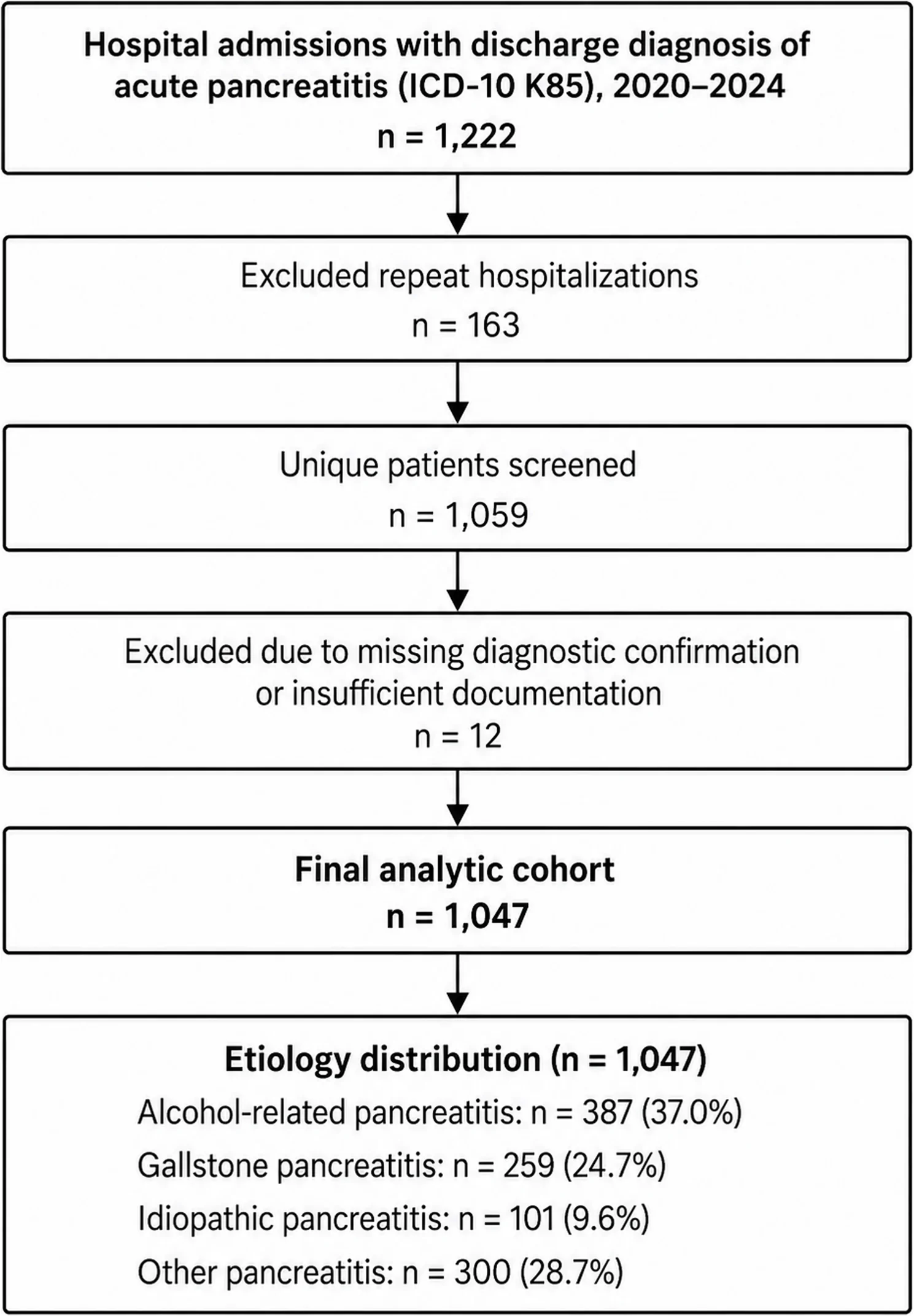
Study cohort selection and etiological distribution of acute pancreatitis

A total of 1,222 admissions were identified. After excluding repeat hospitalizations (*n* = 163), 1,059 unique patients remained. An additional 12 patients were excluded due to insufficient documentation, resulting in 1,047 patients in the final analysis. The study cohort selection process and final etiological distribution are shown in Fig. 1.

Flow diagram showing identification of hospital admissions with discharge diagnosis of acute pancreatitis, exclusion of repeat hospitalizations and cases with missing diagnostic confirmation or insufficient documentation, final analytic cohort, and distribution of etiological categories.

The cohort was predominantly male, with a median age of 50 years (IQR 39–64.5). Alcohol-related pancreatitis was the most common etiology. Median length of stay was 6 days (IQR 4–9), ICU admission was uncommon, and in-hospital mortality was 5.3% (Table 1). Transabdominal ultrasound was performed in 907 patients (86.6%) during the index admission.

**Table 1:** Overall cohort characteristics and outcomes (*n* = 1,047)

Variable		Value
Age, years, median (IQR)		50 (IQR 39–64.5)
Male sex, n (%)		649 (62.0)
Etiology, n (%)	Alcohol	387 (37.0)
	Gallstone	259 (24.7)
	Idiopathic	101 (9.6)
	Other	300 (28.7)
Comorbidities, n (%)	Chronic liver disease	402 (38.4)
	Hypertension	398 (37.6)
	Diabetes mellitus	173 (16.5)
	Heart failure	167 (16.0)
	Chronic kidney disease	70 (6.7)
In-hospital complications, n (%)	Any in-hospital complication	215 (20.5)
	Necrotizing pancreatitis	141 (13.5)
	Infected necrotizing pancreatitis	6 (0.6)
	Walled-off necrosis	2 (0.2)
	Pancreatic pseudocyst	56 (5.3)
	MODS	48 (4.6)
ICU admission, n (%)		15 (1.4)
Length of stay, days, median (IQR)		6 (IQR 4–9)
In-hospital mortality, n (%)		56 (5.3)

Continuous variables are presented as median (interquartile range, IQR) and categorical variables as n (%). *Abbreviations*: *MODS* multiple organ dysfunction syndrome, *ICU* intensive care unit

Regarding diagnostic criteria, clinical pain consistent with acute pancreatitis was documented in approximately 1,016 patients (97.0%), serum lipase and/or amylase elevation ≥ 3 times the upper limit of normal in approximately 928 patients (88.6%), and characteristic imaging findings in approximately 672 patients (64.2%). All three diagnostic components were present in approximately 526 patients (50.2%). Median serum lipase was approximately 1,520 U/L (IQR 510–3,850). Serum amylase was not routinely measured at our institution and was available only for a minority of patients, mainly surgical patients; therefore, amylase values were not summarized because they were considered non-representative.

Among patients categorized as having “other” etiology (*n* = 300), available documentation identified hypertriglyceridemia-associated pancreatitis in 18 patients (1.7% of the total cohort; 6.0% of the “other” category), drug-induced pancreatitis in 15 patients (1.4%; 5.0%), post-ERCP pancreatitis in 11 patients (1.1%; 3.7%), and hypercalcemia-associated pancreatitis in 4 patients (0.4%; 1.3%). The remaining 252 patients (24.1%; 84.0%) were retained as other/nonspecified etiology.

**Fig. 2:**
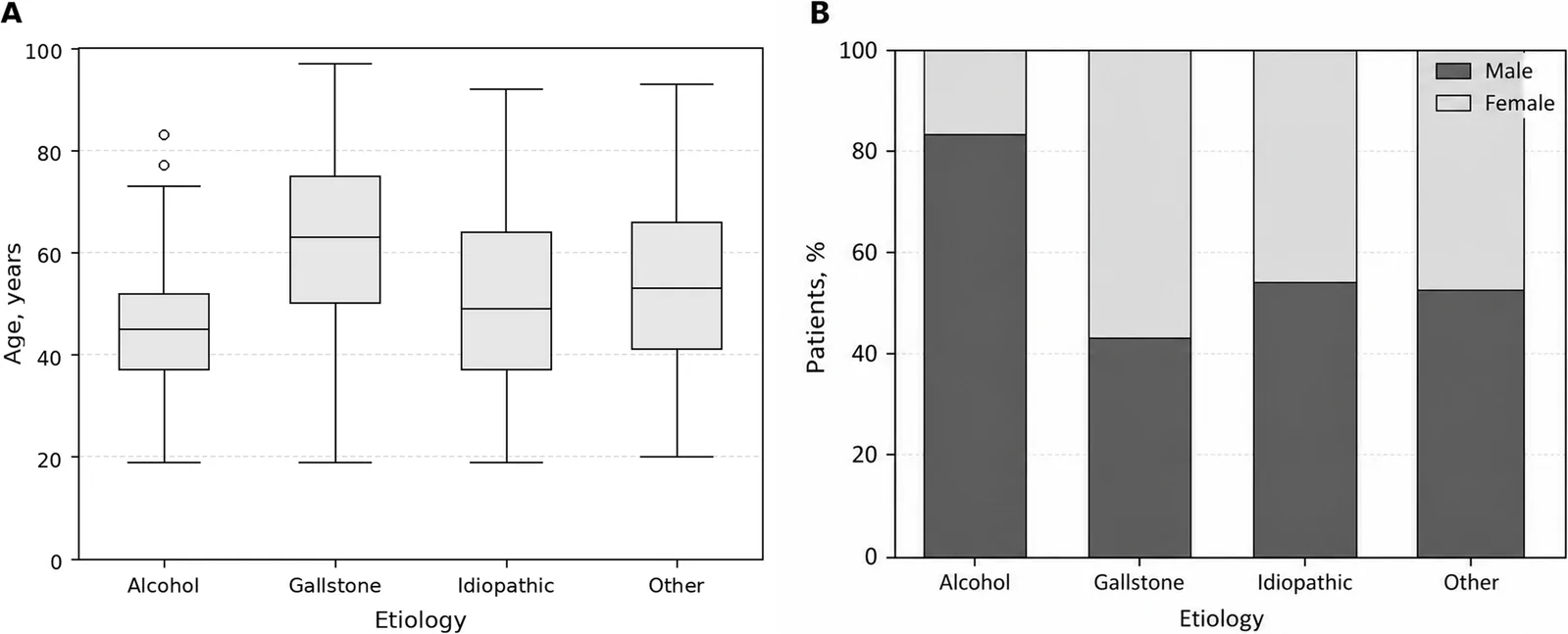
Age and sex distribution by etiology of acute pancreatitis Panel A shows age distribution across etiological groups. Boxes represent the interquartile range, the horizontal line indicates the median, whiskers indicate the range excluding outliers, and circles indicate outliers. Panel B shows proportional sex distribution across etiological groups. Percentages are calculated within each etiological group

Age and sex differed significantly across etiological groups (both *p* < 0.001). Differences were also observed in several comorbidities, including chronic liver disease, heart failure, chronic kidney disease, and diabetes mellitus. Necrotizing pancreatitis also varied between etiologies. Detailed comparisons are shown in Table 2, and age and sex distributions are illustrated in Fig. 2.

**Table 2:** Patient characteristics and complications stratified by etiology

Variable	Alcohol (*n* = 387)	Gallstone (*n* = 259)	Idiopathic (*n* = 101)	Other (*n* = 300)	*p*-value
Age, years (IQR)	45 (37–52)	63 (50–75)	49 (37–64)	53 (42–67)	< 0.001^1^
Male sex, n (%)	323 (83.5)	112 (43.2)	55 (54.5)	159 (53.0)	< 0.001^2^
Comorbidities, n (%)					
Chronic liver disease	209 (54.0)	56 (21.6)	42 (41.6)	95 (31.7)	< 0.001^2^
Hypertension	131 (33.9)	100 (38.6)	44 (43.6)	123 (41.0)	0.148^2^
Diabetes mellitus	51 (13.2)	38 (14.7)	22 (21.8)	62 (20.7)	0.023^2^
Heart failure	28 (7.2)	68 (26.3)	18 (17.8)	53 (17.7)	< 0.001^2^
Chronic kidney disease	5 (1.3)	27 (10.4)	9 (8.9)	29 (9.7)	< 0.001^2^
Local pancreatic complications, n (%)					
Necrotizing pancreatitis	58 (15.0)	19 (7.3)	11 (10.9)	53 (17.1)	0.003^2^
Infected necrotizing pancreatitis	0 (0.0)	1 (0.4)	1 (1.0)	4 (1.3)	0.067^3^
Walled-off necrosis	0 (0.0)	0 (0.0)	0 (0.0)	2 (0.7)	0.327^3^
Pancreatic pseudocyst	27 (7.0)	7 (2.7)	5 (5.0)	17 (5.7)	0.127^2^
Systemic complications, n (%)					
MODS	22 (5.7)	6 (2.3)	4 (4.0)	16 (5.3)	0.204^2^

Continuous variables are presented as median (IQR) and categorical variables as n (%)

*Abbreviations*: *MODS* multiple organ dysfunction syndrome

^1^Kruskal–Wallis test. ^2^Chi-square test. ^3^Fisher’s exact test

Individual systemic complications were additionally identified using ICD-10 diagnostic codes. Acute renal failure (N17) was documented in 42 patients (4.0%), respiratory failure (J96) in 3 patients (0.3%), shock (R57) in 9 patients (0.9%), systemic inflammatory response/sepsis-related systemic response (R65) in 16 patients (1.5%), and sepsis (A41) in 41 patients (3.9%). Overall, MODS was documented in 48 patients (4.6%).

**Fig. 3:**
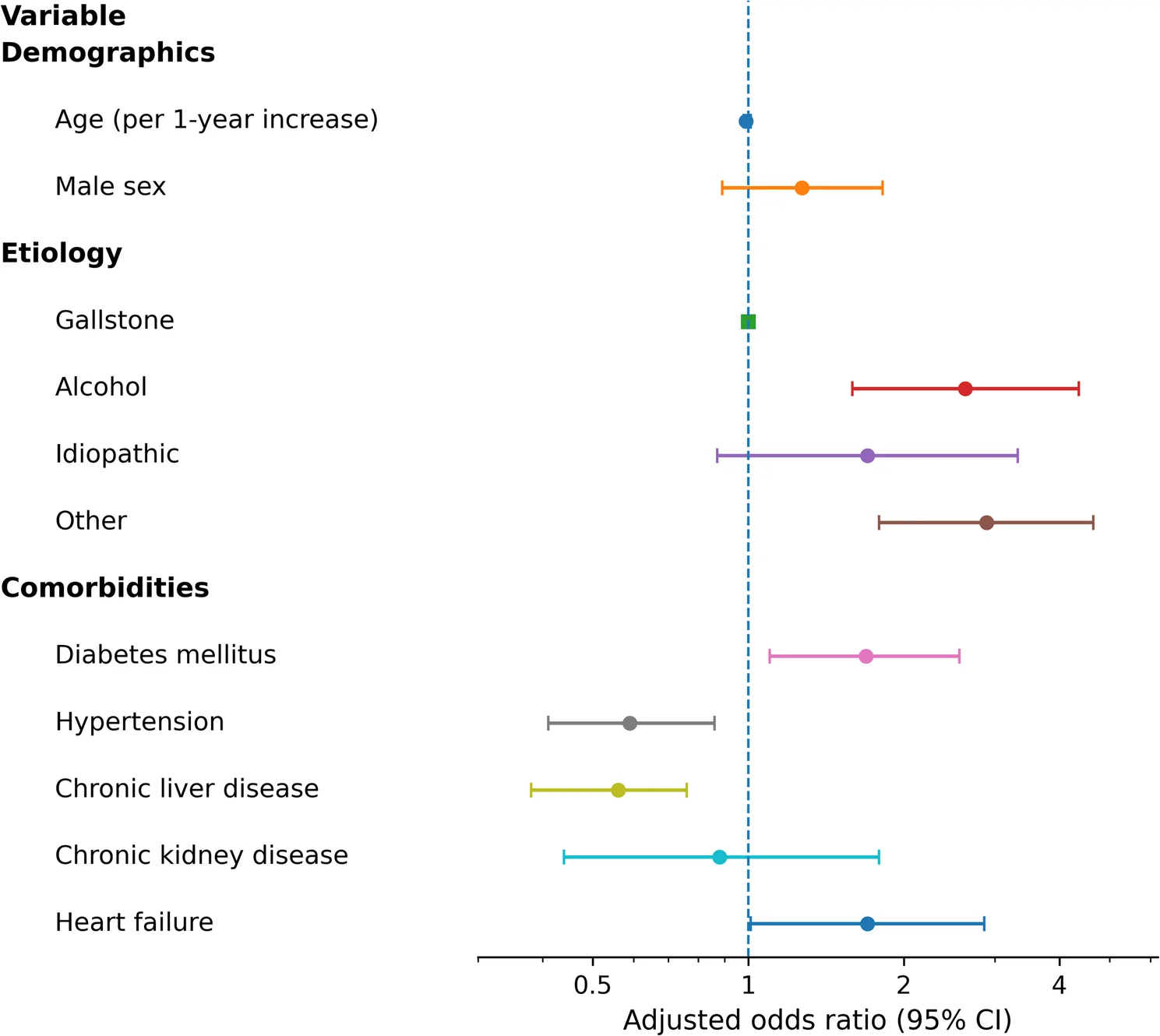
Multivariable logistic regression analysis of factors associated with complications

In multivariable logistic regression, alcohol-related and “other” etiologies were independently associated with higher odds of in-hospital complications compared with gallstone pancreatitis. Diabetes mellitus and heart failure were also associated with increased odds, whereas hypertension and chronic liver disease were associated with lower odds. Overall, 215 patients (20.5%) developed at least one in-hospital complication. Detailed results are presented in Table 3; Fig. 3.

**Table 3:** Multivariable logistic regression analysis of factors associated with complications

Variable		Adjusted OR	95% CI	*p*-value
Age (per 1-year increase)		0.99	0.98–1.01	0.342
Male sex		1.27	0.89–1.82	0.183
Etiology	Gallstone (Reference)	1.00	-	-
	Alcohol	2.63	1.59–4.36	< 0.001
	Idiopathic	1.70	0.87–3.32	0.120
	Other	2.89	1.79–4.65	< 0.001
Comorbidities	Diabetes mellitus	1.69	1.10–2.56	0.013
	Hypertension	0.59	0.41–0.86	0.006
	Chronic liver disease	0.56	0.38–0.76	< 0.001
	Chronic kidney disease	0.88	0.44–1.79	0.726
	Heart failure	1.70	1.01–2.86	0.047

Results are presented as adjusted odds ratios (ORs) with 95% confidence intervals (CIs) from a multivariable logistic regression model. Gallstone pancreatitis served as the reference category

Forest plot showing adjusted odds ratios (ORs) with 95% confidence intervals for demographic characteristics, etiological categories, and comorbidities derived from the multivariable logistic regression model. Gallstone pancreatitis served as the reference etiologic category.

Comparison of admissions during the COVID-19 pandemic period (2020–2021) and post-pandemic period (2022–2024) showed modest differences in etiologic distribution and age, whereas rates of MODS, ICU admission, and discharge status did not differ significantly between periods. Detailed results are presented in Table S2.

In a multivariable model additionally including pandemic-period admission, the primary associations remained unchanged. Pandemic-period admission was not independently associated with complications (adjusted OR 0.77, 95% CI 0.56–1.06; *p* = 0.114; Table S3).

Sensitivity analyses confirmed the robustness of the findings. Excluding pandemic-period admissions (*N* = 614) yielded similar results, although diabetes mellitus was no longer significantly associated with complications (*p* = 0.243) (Table S4). Using a stricter outcome definition (necrotizing pancreatitis, infected necrosis, MODS, or ICU admission; 175 events) did not materially alter the results (Table S5). Combining idiopathic and “other” etiologies also produced consistent estimates, indicating that the findings were not sensitive to etiologic classification (Table S6).

## Discussion

In this retrospective cohort of 1,047 patients hospitalized with acute pancreatitis at a tertiary-center in Latvia, alcohol-related pancreatitis was the most common etiology and predominated in younger male patients, whereas gallstone pancreatitis was associated with older age. Alcohol-related and “other” etiologies were independently associated with higher odds of in-hospital complications compared with gallstone pancreatitis. Despite a measurable complication burden, ICU admission was uncommon. Diabetes mellitus and heart failure were associated with increased risk of complications, whereas hypertension and chronic liver disease showed inverse associations in the adjusted analysis. These findings provide insight into the clinical characteristics and outcomes of acute pancreatitis in a tertiary center cohort from the Baltic region.

Alcohol-related pancreatitis accounted for 37.0% of cases in this cohort, exceeding gallstone pancreatitis, which accounted for 24.7%. Although gallstones are the leading cause of acute pancreatitis in many international cohorts, the relative contribution of biliary and alcohol-related disease varies substantially by region [4, 17, 18]. In a European systematic review, gallstone etiology predominated in southern Europe, whereas alcohol-related etiology was relatively more prominent in eastern and northern Europe, with Latvia, Lithuania, Romania, Hungary, Russia, and Finland among countries in which alcohol-related pancreatitis accounted for a relatively high proportion of cases compared with gallstone pancreatitis [4]. This regional pattern is consistent with our findings and suggests that the predominance of alcohol-related pancreatitis in our tertiary-center cohort is not an isolated observation. Similar geographic heterogeneity has also been reported in older multicountry European data, where alcohol predominated over cholelithiasis in Hungary, while cholelithiasis predominated in southern European countries such as Greece and Italy [19].

The Latvian context further supports this interpretation. Latvia has a high population-level burden of alcohol exposure, with recorded adult alcohol consumption increasing from 9.8 L in 2010 to 12.6 L in 2020 [20]. Alcohol-related conditions contribute substantially to national morbidity and mortality in Latvia, accounting for approximately 6% of deaths and 804.3 disability-adjusted life years per 100,000 population [20]. Broader analyses of alcohol control policies in the Baltic countries and Poland, as well as in former Soviet Union countries, similarly describe persistently high alcohol exposure and alcohol-attributable disease burden in this region [21, 22]. Therefore, the predominance of alcohol-related acute pancreatitis in this cohort is biologically and epidemiologically plausible, particularly given the younger male profile of patients with alcohol-related disease observed in our data [23–25].

Nevertheless, this finding should be interpreted cautiously because the study was retrospective and single-center. We cannot infer the national etiologic distribution of acute pancreatitis or determine whether the background prevalence of gallstone disease is lower in Latvia than in other European regions. We also considered the possibility of missed biliary pancreatitis. In our institution, patients admitted with acute pancreatitis routinely undergo transabdominal ultrasound to assess for gallstones, biliary sludge, cholecystitis, common bile duct dilatation, or other signs of biliary obstruction. This approach is consistent with guideline recommendations that transabdominal ultrasound should be performed in patients with acute pancreatitis to evaluate for biliary etiology, with repeat ultrasound if the initial examination is inconclusive [8, 14]. Patients with clinical or biochemical cholestasis, suspected choledocholithiasis, biliary duct dilatation, or visible stones undergo additional biliary imaging, usually MRI/MRCP, and ERCP is performed when therapeutic duct clearance is indicated. Additional evaluation with MRI/MRCP and/or endoscopic ultrasound (EUS) is recommended when biliary etiology remains uncertain or idiopathic acute pancreatitis is suspected [8, 14, 26]. Therefore, systematic misclassification of gallstone pancreatitis as alcohol-related pancreatitis is unlikely to fully explain the observed etiologic distribution. However, occult microlithiasis, biliary sludge, transient stone passage, incomplete documentation, or cases without definitive biliary imaging may still have led to under-recognition of biliary pancreatitis in a minority of patients [27, 28].

We also considered whether the relatively lower proportion of gallstone pancreatitis could reflect missed biliary disease. This is unlikely to fully explain the observed etiologic distribution, because transabdominal ultrasound was performed in 907 patients (86.6%) and is routinely used in our institution as first-line imaging to evaluate for gallstones, biliary sludge, cholecystitis, common bile duct dilatation, and other signs of biliary obstruction. Patients with suspected choledocholithiasis, cholestatic clinical or biochemical features, biliary duct dilatation, or visible stones undergo additional biliary imaging, usually MRI/MRCP, and ERCP is performed when therapeutic duct clearance is indicated. This diagnostic pathway is consistent with guideline recommendations that transabdominal ultrasound should be performed to evaluate biliary etiology in acute pancreatitis, with additional MRI/MRCP or EUS considered when the etiology remains uncertain [8, 14, 26]. Nevertheless, CT alone may miss radiolucent gallstones, biliary sludge, microlithiasis, or transient common bile duct stones. Therefore, although systematic misclassification of gallstone pancreatitis as alcohol-related pancreatitis is unlikely, occult microlithiasis, transient stone passage, incomplete documentation, or absence of definitive biliary imaging in a minority of patients may have led to under-recognition of biliary etiology [27, 28].

The proportion of cases categorized as “other” etiology in this cohort was relatively high, accounting for 28.7% of patients. In most epidemiologic studies, less common causes, including post-ERCP, drug-induced, traumatic, infectious, metabolic, and other rare etiologies, collectively account for less than 10–15% of acute pancreatitis cases [4, 17]. In our cohort, available documentation allowed partial subclassification of this group, identifying hypertriglyceridemia-associated, drug-induced, post-ERCP, and hypercalcemia-associated pancreatitis. However, most cases in the “other” category could not be reliably reassigned to more specific etiological subgroups retrospectively.

The higher proportion observed in this study likely reflects limitations inherent to retrospective clinical documentation and local discharge-coding practices. Etiologic classification relied on routine electronic medical records, where detailed etiologic work-up, alcohol exposure, biliary findings, metabolic abnormalities, medication exposure, or procedure-related causes may not always have been recorded in a standardized way. Consequently, some cases of alcohol-related, gallstone, or rare-cause pancreatitis may have been classified under nonspecific categories. Institutional differences in diagnostic pathways and documentation practices may also contribute to such classification variability. Therefore, the relatively large “other” group observed in this cohort should be interpreted cautiously as a heterogeneous administrative-clinical category rather than a single biological etiology.

Similarly, idiopathic pancreatitis should be interpreted as “no etiology identified after routine inpatient evaluation” rather than as a definitive etiologic diagnosis. Because this was a retrospective study, we could not confirm that all patients underwent a standardized prospective etiological work-up, including uniform EUS, MRCP, genetic testing, or repeat outpatient evaluation. Therefore, occult biliary disease, microlithiasis, transient stone passage, medication-related causes, metabolic abnormalities, or incomplete documentation may have led to misclassification in a minority of cases.

Consistent with prior studies, alcohol-related pancreatitis predominantly affected younger patients, whereas gallstone pancreatitis was associated with older age [23, 24]. In this cohort, median age was 45 years for alcohol-related disease and 63 years for gallstone pancreatitis. This pattern aligns with epidemiologic evidence showing that alcohol-related pancreatitis typically occurs in younger or middle-aged adults, while the prevalence of gallstone disease increases with age [25, 29, 30]. However, the magnitude of the age difference observed in this cohort appears relatively pronounced. For example, one study reported a smaller age gap between alcohol-related and gallstone pancreatitis (mean ages 37 vs. 43 years) despite similar etiologic categories [25], suggesting that the nearly two-decade difference observed here may reflect a distinct demographic profile within this tertiary center population.

Necrotizing pancreatitis occurred approximately twice as frequently in alcohol-related acute pancreatitis compared with gallstone pancreatitis (15.0% vs. 7.3%). Published data on this association are heterogeneous and partly dependent on how necrosis is defined and assessed. Some studies report higher necrosis rates in alcohol-related disease [31, 32], whereas others have found that the presence and extent of necrosis are independent of etiology [33]. These discrepancies likely reflect differences in study design, imaging practices, severity definitions, and outcome ascertainment. In the present cohort, alcohol-related pancreatitis was associated with higher odds of the composite in-hospital complication endpoint; however, because formal Revised Atlanta severity categories could not be reconstructed and necrosis severity was not assessed in detail, this finding should be interpreted as a difference in recorded adverse in-hospital outcomes rather than definitive evidence of greater disease severity by etiology.

The inverse association between hypertension and chronic liver disease and the risk of in-hospital complications contrasts with previous studies showing that comorbidities are typically associated with worse outcomes in acute pancreatitis [34, 35]. Chronic liver disease in particular has been identified as a risk factor for adverse outcomes in several analyses [36–38]. Therefore, these inverse associations should be interpreted cautiously and should not be considered evidence of a protective effect. In this study, chronic liver disease was defined using routinely recorded ICD-10 diagnoses and available clinical documentation, including cirrhosis, chronic hepatitis, and imaging-based hepatic steatosis/fatty liver disease. As a result, this variable represented a heterogeneous category and was not limited to advanced liver disease or cirrhosis. The inverse association may therefore reflect residual confounding, differences in disease severity within the CLD group, incomplete documentation, coding variability, or model specification rather than a causal biological relationship.

ICU admission occurred in 1.4% of patients, which is lower than rates reported in many observational studies, where approximately 7–10% of patients with acute pancreatitis require intensive care [6, 39, 40]. International guidance generally recommends ICU or intermediary-care admission for patients with acute pancreatitis who develop organ failure, particularly cardiovascular, respiratory, or renal failure [8, 14, 41].

At our institution, however, there is no formal written acute pancreatitis-specific ICU admission protocol. ICU transfer is determined by the treating team, usually in consultation with intensive care specialists, according to clinical deterioration, monitoring requirements, and the need for advanced organ support. In practical terms, patients are usually considered for ICU transfer when they require invasive mechanical ventilation, continuous vasopressor support for persistent hemodynamic instability, renal replacement therapy, or management of progressive multiorgan dysfunction.

Therefore, the low ICU admission rate likely reflects local escalation practices, ward-based management of patients who do not require advanced organ support, and possible resource-related triage differences rather than necessarily indicating a low burden of severe disease. These practical institutional criteria are summarized in Table S1.

This interpretation is consistent with local data describing substantial variability in ICU staffing and workload across Latvian hospitals, reflecting limited specialized intensive care resources [42]. Consequently, ICU admission may be reserved for the most critically ill patients requiring advanced organ support [43].

The discrepancy between the low ICU admission rate and higher in-hospital mortality should also be interpreted cautiously. In this cohort, in-hospital mortality exceeded ICU admission, which is counterintuitive if ICU admission is considered a direct marker of disease severity. Guidelines recommend ICU or intermediary-care admission for patients with acute pancreatitis who develop organ failure, particularly cardiovascular, respiratory, or renal failure [41]. However, ICU utilization is also influenced by institutional resources, bed availability, triage thresholds, comorbidity burden, reversibility of acute illness, and perceived benefit from escalation [44–46]. In hospitalized patients with sudden clinical deterioration, reduced ICU bed availability has been associated with a lower likelihood of ICU admission and a higher likelihood of changes in goals of care [44]. ICU triage studies have also shown that admission decisions may be influenced by advance directives, treatment-limitation decisions, ward location, night-time assessment, and ICU occupancy [45, 46]. Delayed ICU admission among critically ill patients has been associated with increased mortality, further supporting that ICU utilization may not fully capture the burden of severe illness when access or escalation is delayed [47]. Therefore, some deaths in our cohort may have occurred on general wards without formal ICU transfer, particularly among patients with rapid deterioration, advanced comorbidity burden, treatment-limitation decisions, or perceived non-benefit from escalation. ICU admission should therefore not be interpreted as a comprehensive marker of severe acute pancreatitis in this retrospective cohort, and the discordance between ICU utilization and mortality represents an important limitation.

In-hospital mortality in this cohort was 5.3%, which falls within the range reported in contemporary studies of acute pancreatitis, where mortality generally ranges from approximately 3% to 10% depending on disease severity and patient population [3, 4, 18]. Most patients experience an uncomplicated clinical course, whereas mortality is largely concentrated among those who develop organ failure or systemic complications [3, 10].

Admissions during the COVID-19 pandemic period showed modest differences in age and etiologic distribution, but complication rates and mortality remained similar to the post-pandemic period. These findings are consistent with recent studies indicating that the pandemic itself did not substantially alter outcomes in patients without concurrent SARS-CoV-2 infection [48–50].

Future research should further investigate epidemiologic patterns of acute pancreatitis in Baltic and Eastern European populations, particularly the roles of alcohol exposure, comorbidities, and potential genetic predispositions. Multicenter prospective studies integrating clinical and behavioral data will be important for determining whether the patterns observed in this cohort reflect broader regional trends.

This study has several strengths. It addresses a regional evidence gap, as detailed cohort studies of acute pancreatitis in the Baltic region remain limited. The analysis includes a large consecutive cohort of 1,047 patients over a five-year period at a tertiary referral center, enabling a comprehensive evaluation of clinical outcomes. All hospital records were individually reviewed to confirm that diagnostic criteria for acute pancreatitis according to the Revised Atlanta Classification were fulfilled, thereby reducing the risk of diagnostic misclassification. Standardized definitions of complications were applied, improving comparability with prior studies, and multivariable regression with predefined sensitivity analyses was used to ensure the robustness of the findings.

Several limitations should be considered when interpreting these results. First, this was a single-center study conducted at a tertiary referral hospital, which may limit external validity. Second, only index admissions were analyzed, excluding recurrent episodes; while this approach prevented overrepresentation of patients with recurrent pancreatitis, it may underestimate the overall disease burden.

Given the retrospective design, etiologic classification and comorbidity data depended on the accuracy and completeness of electronic medical records and may therefore be subject to information bias and misclassification. Chronic liver disease was analyzed as a broad comorbidity category that included cirrhosis, chronic hepatitis, and imaging-based hepatic steatosis/fatty liver disease; therefore, we could not assess whether cirrhosis or advanced liver disease specifically was associated with adverse outcomes. In particular, the relatively large proportion of cases categorized as “other” etiology may reflect limitations in retrospective documentation and local discharge-coding practices, as detailed etiologic work-up or clear documentation of alcohol use, biliary disease, metabolic abnormalities, medication exposure, or procedure-related causes was not consistently available in a standardized format. Consequently, some cases of alcohol-related, gallstone, or rare-cause pancreatitis may have been classified under nonspecific categories. Similarly, idiopathic pancreatitis should be interpreted as no etiology identified after routine inpatient evaluation rather than after a standardized prospective etiological work-up with uniform EUS, MRCP, genetic testing, or repeat outpatient reassessment. Although ultrasound was performed in most patients, the retrospective design did not allow complete reconstruction of biliary workup in all cases; therefore, occult microlithiasis, transient stone passage, or incomplete documentation may have resulted in under-recognition of biliary etiology in a minority of patients.

Severity categories according to the Revised Atlanta Classification are not routinely recorded in the electronic medical records at our institution and could not be reliably reconstructed. This is an important limitation because standardized severity classification is central to interpreting outcomes in acute pancreatitis. Clinical management in routine practice was based on the patient’s clinical trajectory, presence of local or systemic complications, organ dysfunction, need for imaging or intervention, and requirement for escalation of care rather than formal prospective assignment of mild, moderately severe, or severe acute pancreatitis categories. The primary endpoint, which combined local pancreatic and systemic complications, should therefore be interpreted as a broad indicator of adverse in-hospital course rather than a direct surrogate for standardized severity classification. Although local pancreatic complications and systemic complications were reported separately, the primary adjusted model used a composite complication endpoint because individual severe outcomes were infrequent. This endpoint should therefore be interpreted as a broad marker of adverse in-hospital course and not as implying clinical equivalence between local and systemic complications.

In addition, the retrospective design did not allow reliable assessment of the severity, extent, or anatomical distribution of pancreatic necrosis from imaging. We therefore could not classify necrosis by percentage of gland involvement, extrapancreatic extension, or imaging-based severity indices. This limits interpretation of local complications, because necrotizing pancreatitis represents a heterogeneous entity with variable clinical severity. As a result, etiologic differences across formal severity categories could not be assessed.

The number of severe complications and ICU admissions was relatively small, which may reflect institutional triage practices and limits statistical power for the evaluation of rare outcomes. The discordance between ICU admission and in-hospital mortality indicates that ICU admission may incompletely capture severe disease in this cohort, particularly if some deaths occurred without formal ICU transfer because of rapid deterioration, local escalation thresholds, limited ICU access, or treatment-limitation decisions. Because individual systemic complications were identified retrospectively from routinely recorded diagnostic codes and clinical documentation, the reported frequencies may underestimate the true occurrence of organ dysfunction during hospitalization.

Treatment-related variables, including fluid resuscitation strategy, timing and route of nutritional support, analgesic management, and other aspects of inpatient treatment, were not analyzed. These variables were beyond the epidemiologic focus of the present study and could not be captured consistently or reliably from retrospective electronic medical records.

No correction for multiple comparisons was applied to the exploratory univariable comparisons in Table 2; therefore, marginal p-values from these analyses should be interpreted cautiously. Finally, the analysis was restricted to in-hospital outcomes and does not capture post-discharge events or long-term prognosis.

## Conclusion

In this tertiary-center cohort from Latvia, alcohol-related acute pancreatitis was the predominant etiology and was associated with higher odds of a composite in-hospital complication endpoint compared with gallstone pancreatitis. Because formal severity categories could not be reliably reconstructed and the primary outcome combined local and systemic complications, these findings should be interpreted as evidence of differences in recorded adverse in-hospital outcomes rather than definitive differences in acute pancreatitis severity. The results suggest that alcohol-related disease may contribute substantially to the acute pancreatitis hospital burden in this setting and support the need for improved etiologic identification and prevention strategies targeting alcohol-related disease.

## Supplementary Information

Supplementary Material 1: File S1. Completed STROBE checklist for cohort studies.

Supplementary Material 2: Table S1. Practical institutional indications for ICU transfer in acute pancreatitis.

Supplementary Material 3: Table S2. Comparison of acute pancreatitis admissions during the COVID-19 pandemic period and post-pandemic period.

Supplementary Material 4: Table S3. Multivariable logistic regression model including pandemic-period admission as a predictor of in-hospital complications.

Supplementary Material 5: Table S4. Sensitivity analysis excluding pandemic-period admissions (2022–2024 only; *n* = 614; events = 136).

Supplementary Material 6: Table S5. Sensitivity analysis using a stricter complication endpoint (*n* = 1,047; events = 175).

Supplementary Material 7: Table S6. Sensitivity analysis combining idiopathic and other etiologies (*n* = 1,047; events = 215).

## Data Availability

The data supporting the findings of this study are available from the corresponding author upon reasonable request.

## Abbreviations

AP: Acute pancreatitis
CI: Confidence interval
ERCP: Endoscopic retrograde cholangiopancreatography
EUS: Endoscopic ultrasound
ICD-10: International Classification of Diseases, 10th Revision
ICU: Intensive care unit
IQR: Interquartile range
MODS: Multiple organ dysfunction syndrome
MRCP: Magnetic resonance cholangiopancreatography
MRI: Magnetic resonance imaging
OR: Odds ratio
SPSS: Statistical Package for the Social Sciences
STROBE: Strengthening the Reporting of Observational Studies in Epidemiology
