# Supplementary material for: Clinical characteristics, etiology, and outcomes of acute pancreatitis at a tertiary referral center in Latvia: a retrospective cohort study
Source: BMC Gastroenterol. 2026 Jun 27;26:561. doi: 10.1186/s12876-026-05064-1 (PMC13576186; doi:10.1186/s12876-026-05064-1)
Supplement: Supplementary file 1 [file 12876_2026_5064_MOESM1_ESM.pdf]

Completed STROBE Checklist for Cohort Studies

Clinical characteristics, etiology, and outcomes of acute pancreatitis at a tertiary referral center in Latvia: a retrospective cohort study

STROBE Statement - Checklist of items that should be included in reports of cohort studies.

The final column gives the page and exact manuscript line numbers where each item is addressed in the revised manuscript. Items that are not applicable to this retrospective in-hospital cohort are marked as not applicable.

**Section**	**Item No.**	**Location in revised manuscript**
**Title and abstract**	1a	Title page, p. 1, lines 1-2.
	1b	Abstract, p. 2-3, lines 10-39.
**Introduction**	2	Background, p. 3-4, lines 42-69.
	3	Objective statement, p. 4, lines 67-69.
**Methods**	4	Study design, p. 4, lines 71-77.
	5	Study setting, p. 4, lines 71-77.
	6a	Study population and eligibility criteria, p. 4-5, lines 78-89.
	6b	Not applicable.
	7	Diagnostic criteria and clinical variables, p. 5, lines 99-102; 113-130.
	8	Data sources and definitions, p. 5, lines 91-99.
	9	Index admission only and exclusion criteria, p. 5, lines 86-89.
	10	Consecutive adult hospitalized patients during 2020-2024, p. 8-9, lines 169-178.
	11	Descriptive statistics and normality testing, p. 7, lines 132-139.
	12a	Statistical analysis, p. 7-8, lines 131-167.
	12b	Pandemic-period comparison, p. 8, lines 153-155.
	12c	Missing key variables handled during cohort selection and final dataset statement, p. 8, lines 164-167.
	12d	Not applicable.
	12e	Sensitivity analyses described in Methods, p. 7-8, lines 140-167.
**Results**	13a	Cohort derivation, p. 8-9, lines 168-178.
	13b	Reasons for exclusion: repeat hospitalizations and insufficient documentation, p. 8, lines 168-172.
	13c	Flow diagram, Fig. 1, p. 9, lines 173-178.
	14a	Overall cohort characteristics, Table 1, p. 9-10, lines 179-187.
	14b	Missing key variables addressed during cohort selection p. 8, lines 168-172.
	14c	Not applicable.
	15	Outcome events in Table 1, p. 9-10, lines 184-187.
	16a	Unadjusted comparisons by etiology, Table 2, p. 11-12, lines 212-215.
	16b	Not applicable for the main multivariable model.

	16c	Outcome event counts and percentages are provided in Table 1, p. 9-10, lines 184-187, and Table 2, p. 11-12, lines 212-215.
	17	Pandemic-period analyses, p. 15, lines 238-244; sensitivity analyses, p. 15, lines 245-251; Supplementary Tables S2-S6, pp. 33-37, lines 740-771.
**Discussion**	18	Opening Discussion summary, p. 15, lines 252-262.
	19	Limitations section, p. 23-25, lines 444-504.
	20	Discussion interpretation and comparison with prior literature, p. 16-25, lines 263-504; conclusion, p. 25, lines 505-514.
	21	Single-center external validity limitation, p. 23, lines 443-448; regional interpretation and future multicenter research, p. 22, lines 430-434; in-hospital-only limitation, p. 25, lines 503-504.
**Other information**	22	Funding statement, p. 27, lines 547-548. No funding was received.
