# Supplementary material for: Clinical characteristics, etiology, and outcomes of acute pancreatitis at a tertiary referral center in Latvia: a retrospective cohort study
Source: BMC Gastroenterol. 2026 Jun 27;26:561. doi: 10.1186/s12876-026-05064-1 (PMC13576186; doi:10.1186/s12876-026-05064-1)
Supplement: Supplementary file 2 [file 12876_2026_5064_MOESM2_ESM.docx]

**Table S1**. Practical institutional indications for ICU transfer in acute pancreatitis.

| **Clinical condition** | **Practical indications for ICU transfer** |
| --- | --- |
| **Respiratory failure** | Requirement for invasive mechanical ventilation, intubation, or respiratory support exceeding ward capacity |
| **Hemodynamic instability/shock** | Persistent hypotension despite fluid resuscitation or need for continuous vasopressor support |
| **Severe renal failure** | Acute kidney injury requiring urgent renal replacement therapy or dialysis-level support |
| **Multiorgan dysfunction** | Progressive deterioration involving ≥2 organ systems requiring continuous monitoring or advanced organ support |
| **Other clinician-determined need** | ICU transfer based on treating physician and intensive care specialist assessment when monitoring or treatment requirements exceed ward capacity |

These are practical institutional criteria rather than formal written acute pancreatitis-specific ICU admission guidelines. ICU transfer is determined case by case according to clinical trajectory, organ support requirements, monitoring needs, ward capacity, and available ICU resources.
