# Supplementary material for: Clinical characteristics, etiology, and outcomes of acute pancreatitis at a tertiary referral center in Latvia: a retrospective cohort study
Source: BMC Gastroenterol. 2026 Jun 27;26:561. doi: 10.1186/s12876-026-05064-1 (PMC13576186; doi:10.1186/s12876-026-05064-1)
Supplement: Supplementary file 3 [file 12876_2026_5064_MOESM3_ESM.docx]

**Table S2**. Comparison of acute pancreatitis admissions during the COVID-19 pandemic period and post-pandemic period.

| **Variable** | **Pandemic period, 2020-2021 (n = 433)** | **Post-pandemic period, 2022-2024 (n = 614)** | **p-value** |
| --- | --- | --- | --- |
| **Age, years, median (IQR)** | 52 (43-66) | 48 (38-62) | <0.001^1^ |
| **Etiology, n (%)** |  |  | 0.001^2^ |
| Gallstone | 127 (29.3) | 132 (21.5) |  |
| Alcohol | 152 (35.1) | 235 (38.3) |  |
| Idiopathic | 27 (6.2) | 74 (12.1) |  |
| Other | 127 (29.3) | 173 (28.2) |  |
| **MODS, n (%)** | 22 (5.1) | 26 (4.2) | 0.519^2^ |
| **ICU admission, n (%)** | 6 (1.4) | 9 (1.5) | 0.914^2^ |
| **Discharge status, n (%)** |  |  | 0.751^2^ |
| In-hospital death | 23 (5.3) | 33 (5.4) |  |
| Discharged home | 403 (93.1) | 567 (92.3) |  |
| Transferred | 7 (1.6) | 14 (2.3) |  |

Continuous variables are presented as median (interquartile range, IQR), and categorical variables as n (%). ^1^Mann-Whitney U test. ^2^Chi-square test. Abbreviations: ICU, intensive care unit; MODS, multiple organ dysfunction syndrome.
