# Supplementary material for: Clinical characteristics, etiology, and outcomes of acute pancreatitis at a tertiary referral center in Latvia: a retrospective cohort study
Source: BMC Gastroenterol. 2026 Jun 27;26:561. doi: 10.1186/s12876-026-05064-1 (PMC13576186; doi:10.1186/s12876-026-05064-1)
Supplement: Supplementary file 4 [file 12876_2026_5064_MOESM4_ESM.docx]

**Table S3**. Multivariable logistic regression model including pandemic-period admission as a predictor of in-hospital complications.

| **Variable** | **Adjusted OR** | **95% CI** | **p-value** |
| --- | --- | --- | --- |
| **Age, per 1-year increase** | 0.995 | 0.983-1.007 | 0.383 |
| **Male sex** | 1.275 | 0.893-1.822 | 0.182 |
| **Etiology** |  |  | <0.001 |
| Gallstone | Reference | - | - |
| Alcohol | 2.593 | 1.566-4.293 | <0.001 |
| Idiopathic | 1.600 | 0.816-3.137 | 0.171 |
| Other | 2.846 | 1.765-4.588 | <0.001 |
| **Diabetes mellitus** | 1.759 | 1.156-2.678 | 0.008 |
| **Hypertension** | 0.586 | 0.402-0.854 | 0.005 |
| **Chronic liver disease** | 0.560 | 0.397-0.789 | <0.001 |
| **Chronic kidney disease** | 0.847 | 0.418-1.718 | 0.645 |
| **Heart failure** | 1.739 | 1.031-2.933 | 0.038 |
| **Pandemic-period admission, 2020-2021** | 0.770 | 0.557-1.064 | 0.114 |

Results are presented as adjusted odds ratios (ORs) with 95% confidence intervals (CIs). The dependent variable was the composite in-hospital complication endpoint. Gallstone pancreatitis, female sex, absence of comorbidity, and post-pandemic admission were used as reference categories. The model included age, sex, etiology, diabetes mellitus, hypertension, chronic liver disease, chronic kidney disease, heart failure, and pandemic-period admission.
