# Supplementary material for: Clinical characteristics, etiology, and outcomes of acute pancreatitis at a tertiary referral center in Latvia: a retrospective cohort study
Source: BMC Gastroenterol. 2026 Jun 27;26:561. doi: 10.1186/s12876-026-05064-1 (PMC13576186; doi:10.1186/s12876-026-05064-1)
Supplement: Supplementary file 6 [file 12876_2026_5064_MOESM6_ESM.docx]

**Table S5**. Sensitivity analysis using a stricter complication endpoint (n = 1,047; events = 175).

| **Variable** | **Adjusted OR (95% CI)** | **p-value** |
| --- | --- | --- |
| **Etiology: gallstone** | Reference | - |
| **Age (per 1-year increase)** | 1.00 (0.99-1.01) | 0.917 |
| **Male sex** | 1.29 (0.88-1.90) | 0.190 |
| **Alcohol** | 2.31 (1.34-4.00) | 0.003 |
| **Idiopathic** | 1.82 (0.89-3.71) | 0.099 |
| **Other** | 2.90 (1.73-4.84) | <0.001 |
| **Diabetes mellitus** | 1.98 (1.28-3.06) | 0.002 |
| **Hypertension** | 0.67 (0.45-0.99) | 0.047 |
| **Chronic liver disease** | 0.55 (0.38-0.80) | 0.002 |
| **Chronic kidney disease** | 0.62 (0.28-1.38) | 0.240 |
| **Heart failure** | 1.42 (0.81-2.48) | 0.218 |

Results are presented as adjusted odds ratios (ORs) with 95% confidence intervals (CIs) from multivariable logistic regression. Gallstone pancreatitis served as the reference etiologic category. Models were adjusted for age, sex, etiology, diabetes mellitus, hypertension, chronic liver disease, chronic kidney disease, and heart failure. The stricter endpoint was defined as necrotizing pancreatitis, infected necrosis, MODS, or ICU admission.
